# Streamlined assembly of cloning and genome editing vectors for genus *Clostridium*

**DOI:** 10.1016/j.isci.2023.107484

**Published:** 2023-07-28

**Authors:** Tom S. Bailey, Philip Hittmeyer, Yanchao Zhang, Aleksandra M. Kubiak

**Affiliations:** 1 GROW - School for Oncology and Reproduction, Faculty of Health and Medical Life Sciences, Maastricht University, Maastricht, Limburg, the Netherlands; 2 Exomnis Biotech BV, Oxfordlaan, Maastricht, Limburg, the Netherlands

**Keywords:** Molecular Genetics, Biotechnology, Microbial biotechnology

## Abstract

Reported herein is a new set of vectors designed to streamline molecular cloning and genome editing by exploiting modern cloning methods. The new vectors build on the existing pMTL8000 vectors that have been a staple of *Clostridium* research for more than a decade. The introduction of two pairs of type IIS restriction sites flanking an insulated multiple cloning site in both a cloning vector and a CRISPR-Cas9 gene editing vector enables plasmid construction in a “one-pot” reaction, avoiding the more laborious steps of conventional cloning. A synthetic *lacZα* expression cassette introduced between the cloning sites enables visual detection of background colonies. In addition, distinct selection markers on each vector permit selection of the desired clones according to antibiotic resistance. An example of strain development using the new vectors is demonstrated.

## Highlights

• One-pot cloning with 97%–99% accuracy
• One-pot transfer of subcloned expression cassettes to a genome editing vector
• Transciptionally insulated cloning sites precludes the need to clone terminators
• The new vectors enable standardized cloning in tubes or microplates

## Graphical abstract

**Figure:**
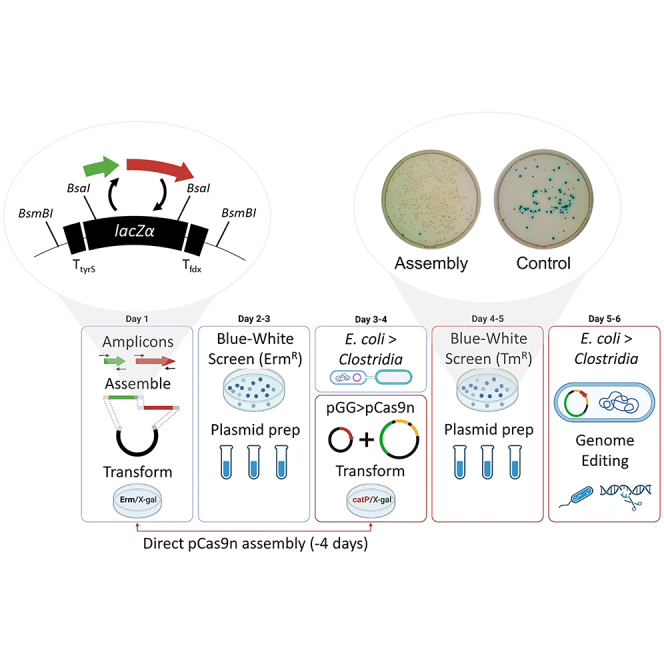

## Introduction

Interest in species of genus *Clostridium* has increased considerably over the last 20 years. While early research efforts focused on pathogenic species, more recent studies have highlighted the ubiquity of clostridia, both in the environment and in mammalian hosts.^1^^,^^2^ In addition, a rapidly expanding list of species relevant to industrial production of solvents, biofuels, and chemical precursors has attracted attention, aided by increasing demand for fossil fuel alternatives.^3^^,^^4^ This enthusiasm is reflected in the literature, with publications containing “Clostridium” in their title increasing from ∼300 per year in the 2000s to a pre-pandemic peak of ∼1300 per year.

The community of researchers that study *Clostridium* species have relied heavily on the pMTL8000 vectors, a series of modular plasmids designed to be easily adapted to the species of interest and the needs of the experiment. These vectors have been a major facilitator for research in this field, enabling gene knock-outs and complementation studies as well as overexpression of native or heterologous proteins, among other uses. Published in 2009, the pMTL8000 vectors functioned as both cloning and expression vectors that could be shuttled between *E. coli* cloning strains and the *Clostridium* species of interest. Conserved restriction sites between functional modules, consisting of a gram-positive replicon, selection marker, gram-negative replicon, and “application-specific” module enabled a simple method for adapting the vector to different clostridia and different purposes. These vectors have spawned several mutagenesis systems, including intron-directed gene disruption,^5^ a mariner transposon system,^6^^,^^7^^,^^8^^,^^9^ an allelic exchange system,^10^^,^^11^^,^^12^ and, most recently, multiple versions of a CRISPR-Cas system.^13^^,^^14^^,^^15^^,^^16^^,^^17^^,^^18^

In parallel to these advances, a number of new, commercially available cloning methods have emerged, including Gibson assembly and its derivatives^19^^,^^20^ and golden gate assembly (GGA).^21^ Common to all of these techniques are a simplified cloning process, typically involving fewer steps and fewer reagents, and the possibility to clone without “scars” (restriction enzyme recognition sequences). GGA has a number of distinct advantages over other techniques. Remarkably complex assemblies are possible using this method. A recent publication demonstrated that precise selection of fusion sites to maximize ligation fidelity enabled assembly of a 40 kb phage genome from 52 fragments.^22^ On a practical level, only two enzymes (a restriction enzyme and DNA ligase) are required for GGA, and the inherent simplicity and efficiency of the reaction make this method more accessible for students. Plasmid construction using GGA does require an upfront effort to develop suitable vectors, that is, addition of the desired type IIS restriction sites in the correct orientation and removal of any existing recognition sequences outside of the multiple cloning site (MCS) (domestication). An example of this is the start-stop system, which uses 3-base cutting type IIS restriction enzymes to construct units from promoters, UTRs, coding sequences, and terminators. By assigning different enzymes to each “assembly level”, large combinatorial assemblies, such as the enzymes of a metabolic pathway, can be constructed.^23^

This paper introduces two new vectors: one for cloning and expression and one for genome editing. Both are based on pMTL8000 plasmids, or their derivatives, and both maintain most of the conserved cloning sites of their forerunners for easy exchange of backbone modules. The central change in both vectors is the creation of a cloning site that is compatible with BsaI-based GGA and blue-white screening using Xgal. The introduction of additional type IIS sites, flanking the cloning site, enables transfer of “subcloned” expression cassettes from cloning vector to gene editing vector in a second GGA reaction. As demonstrated in this paper, the gene editing vector can be “pre-targeted” to a known integration site, for which a guide RNA (gRNA) and repair cassette have been validated previously. The results presented here demonstrate that these vectors can support a fast, cost-effective, and user-friendly method for construction of expression and gene editing plasmids for use in *Clostridium* species.

## Results

### Vector construction

**Figure 1:**
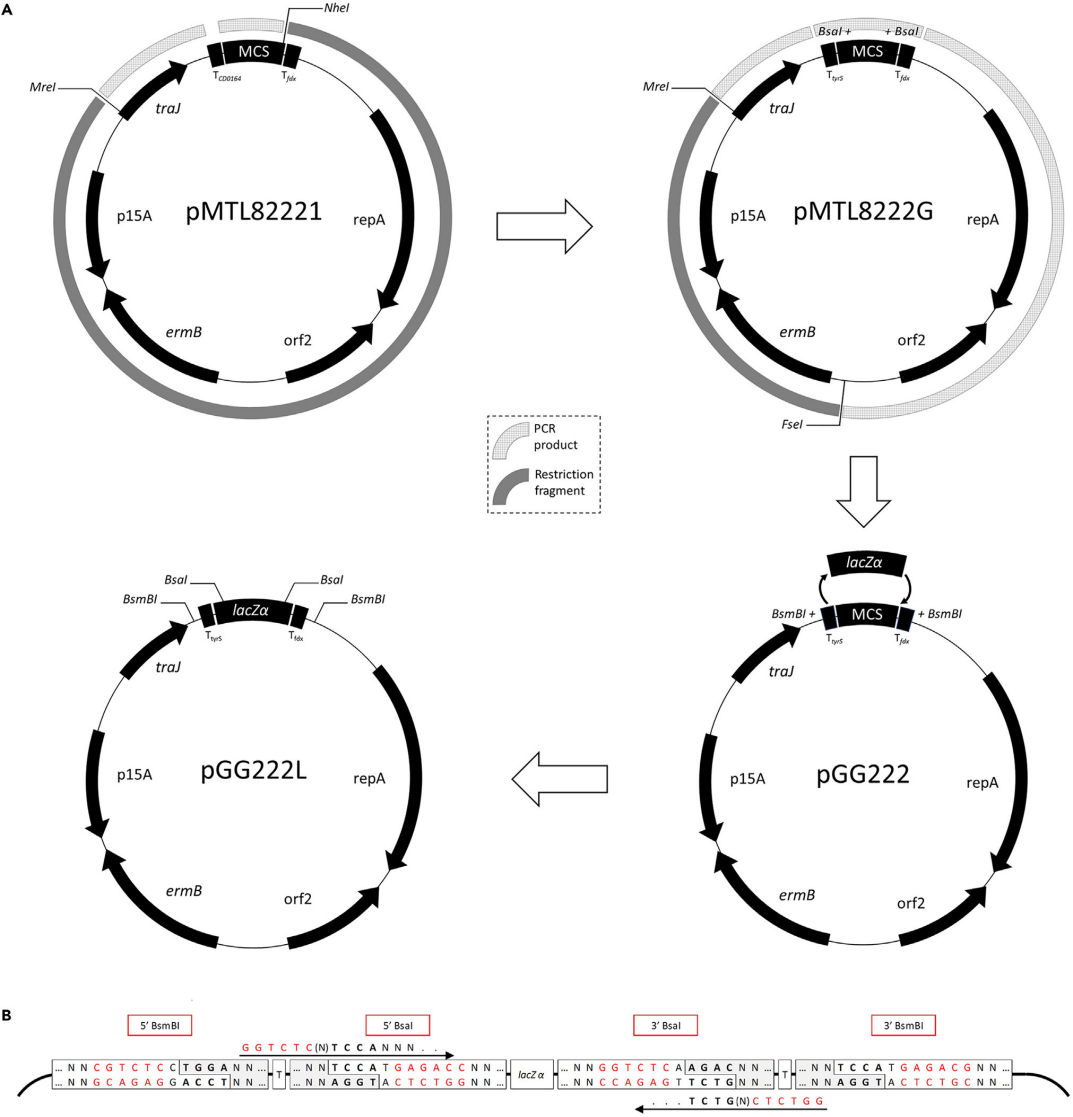
Construction of the pGG222L cloning vector and amplicon primer design Pairs of type IIS restriction sites were added to the original vector via primer tails in two sequential steps (A). The BsaI sites are flanked by characterized terminators to ensure insulated expression. The BsmBI sites flank the terminators so that the entire insulated expression cassette can be transferred to the second vector. BsaI sites can be incorporated into amplicons that are to be assembled into vector pGG222L via primer tails (B). The orientation of the BsaI sites is important to ensure correct Golden Gate assembly.

A number of modifications were made to vector pMTL82221 to support the function of the final vector, pGG222L. The 5′ MCS terminator was replaced by the validated tyrosyl-tRNA synthetase (tyrS) terminator from Bacillus subtilis.^24^ To enable blue-white screening of transformants (blue representing undigested vector), a synthetic *lacZα* coding sequence was added in between the *tyrS* and *C. pasteurianum* ferredoxin (*fdx*) terminators, flanked by BsaI recognition sites. Up- and downstream of the terminators, an additional pair of type IIS restriction sites (BsmBI) were incorporated. The BsaI sites are oriented so that the recognition sequence remains on the empty MCS after cleavage, while the BsmBI sites are oriented to ensure that the recognition sequence remains on the vector backbone. The four-base sticky ends that result from cleavage at these locations were selected based on several factors: avoiding conflict with other cut sites on the vector, the availability of sequences in the original vector, and maximizing ligation efficiency and fidelity (determined using the NEB Ligase Fidelity tool - https://ligasefidelity.neb.com/). The sequence of the final vector with annotations is available from NCBI GenBank (GenBank: OQ302270). A detailed description of the cloning process is provided in the STAR Methods section. Figure 1 is a schematic representation of the process and shows the arrangement and orientation of the cloning sites in the final vector. 5' primer sequences for cloning fragments at the BsaI cloning sites are indicated.

**Figure 2:**
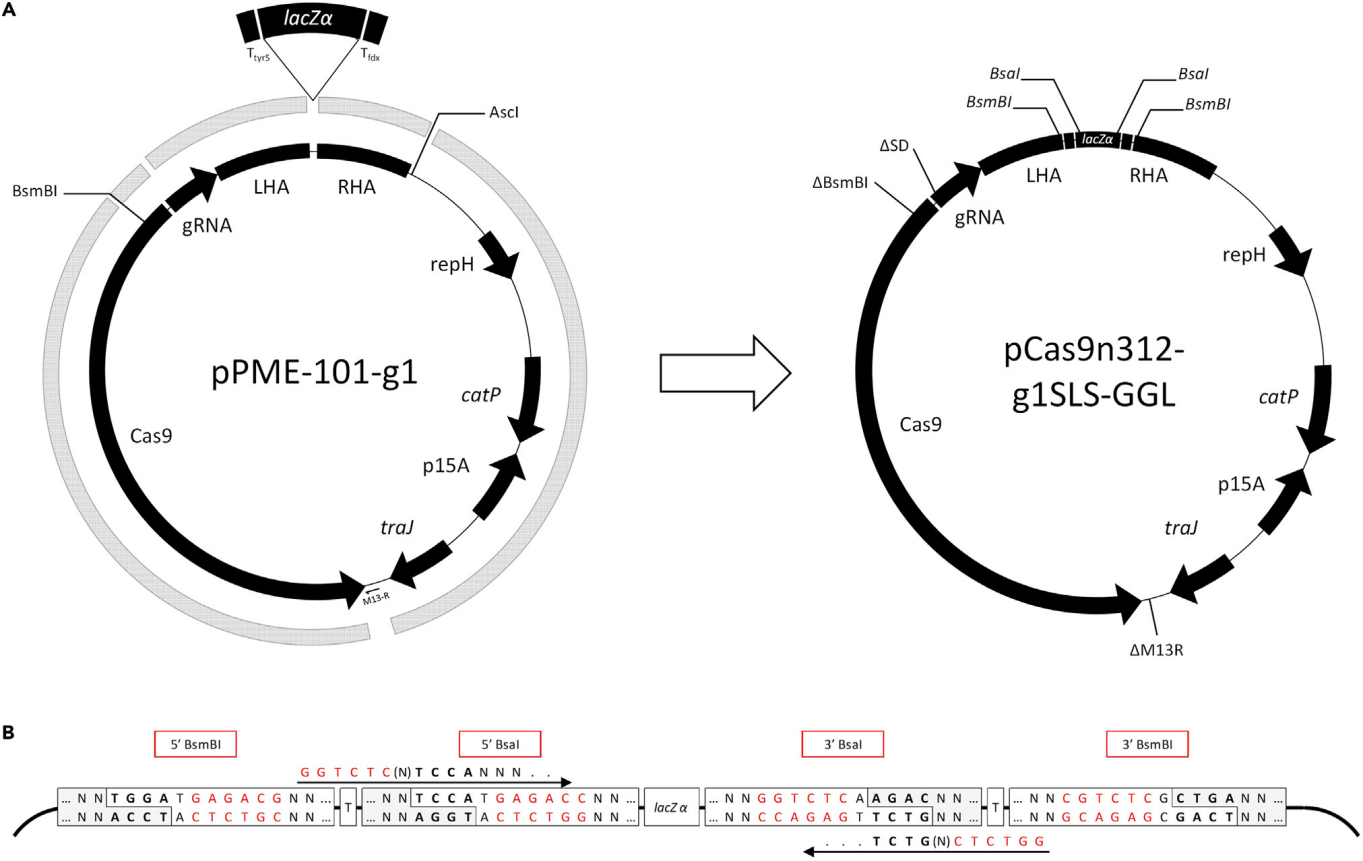
Construction of the gene editing vector and amplicon primer design Five fragments were created by PCR using the previously reported pPME-101-g1 vector as template (A). Sequences were introduced or eliminated through primer tails and selective primer annealing, respectively. The Shine-Dalgarno sequence at the junction between the promoter and coding sequencing of the gRNA and the sequence downstream of the Cas9 gene, corresponding to the M13-R primer annealing site and the CD0164 terminator, were eliminated. A validated terminator was introduced downstream of the Cas9 gene, and the insulated *lacZα* cloning site with flanking BsmBI sites was added between the SLS homology arms. The sequence and orientation of the type IIS restriction sites are shown (B). Primers used to produce amplicons to be cloned at the BsaI sites must contain the 5′ sequences shown (B).

The gene editing vector was derived from the plasmid reported previously.^25^ Alongside the addition of an MCS within the repair cassette, several modifications were made to the CRISPR-Cas9 vector through primer design that excluded sequences or added them through non-annealing primer tails. The previous terminator and M13R primer site downstream of the Cas9 gene were removed and replaced with the validated terminator from the *Lactococcus lactis pepN* gene. The BsmBI restriction site present in the native Cas9 coding sequence was removed via the synonymous codon change, CGT to AGA, and the Shine-Dalgarno sequence of the *C. acetobutylicum* arabinose sugar–proton symporter promoter (P*araE*) was deleted. The full name of the resultant vector is pCas9n312-g7SLS-GGL. The sequence of the final vector with annotations is available from NCBI GenBank (GenBank: OQ302271). A detailed description of the cloning steps to produce pGG222L and pCas9n312-g7SLS-GGL is provided in the STAR Methods section. Figures 1 and 2 are schematic representations of the cloning process for pGG222L and pCas9n312-g7SLS-GGL, respectively, and display the arrangement and orientation of the cloning sites in the final vectors. 5' primer sequences for cloning fragments at the BsaI cloning sites of each vector are indicated.

### Assembly reactions

**Figure 3:**
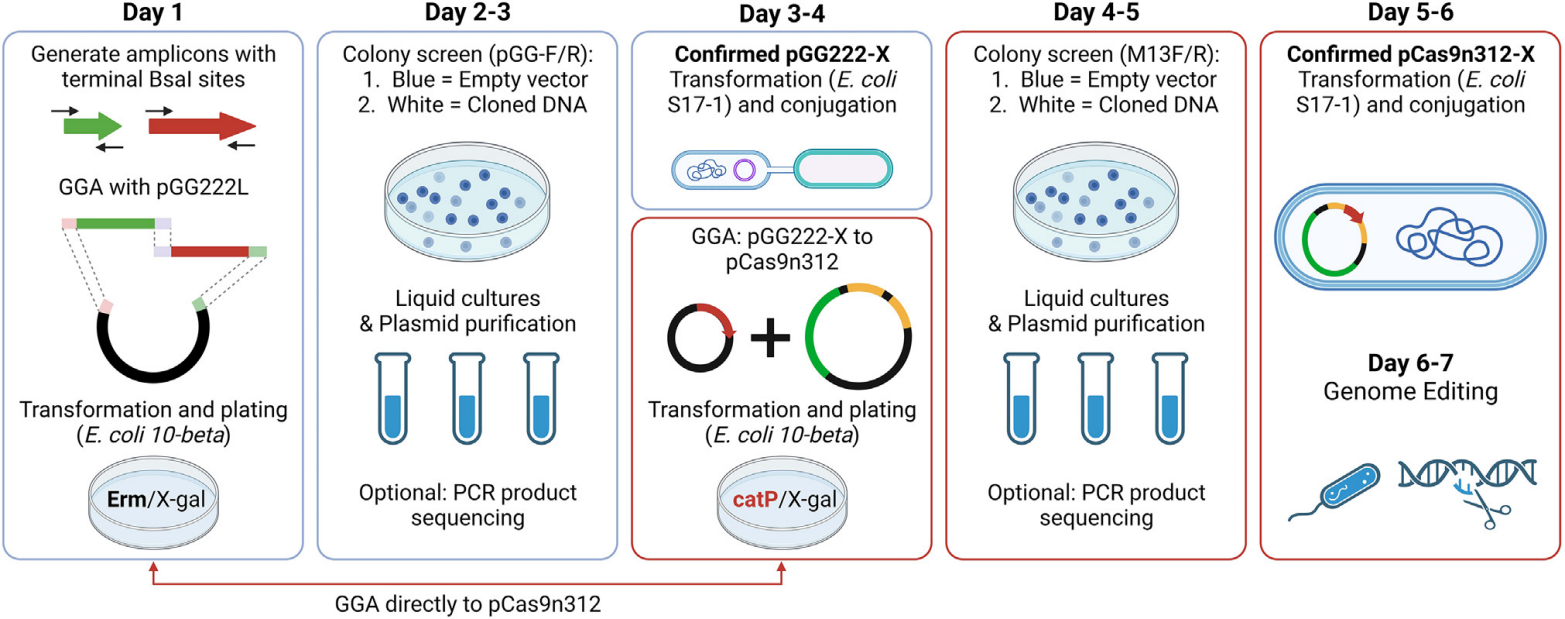
The complete workflow for plasmid assembly and genome editing using the pGG222L and pCas9n312-g7SLS-GGL vectors Screening for correct assemblies is based on antibiotic resistance and white colonies on X-gal plates. Cloning via pGG222L (blue outlines) enables a library of variants to be produced before selection of a limited number for integration, but integrants can be achieved in a shorter time by cloning amplicons directly into the Cas9 vector (red outlines).

**Figure 4:**
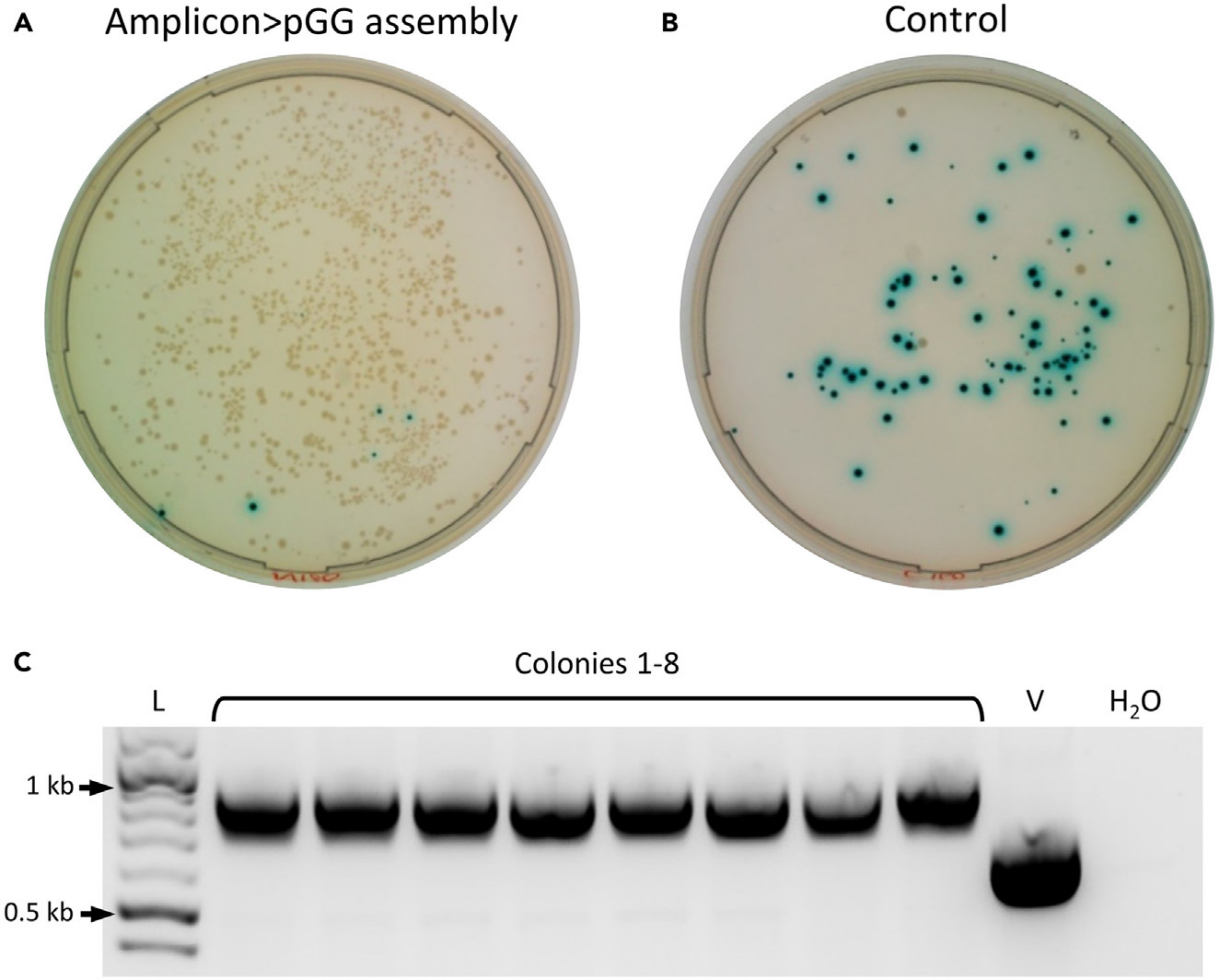
Transformation and PCR screening of the pGG-P*fdx*-NTR assembly Amplicons of the P*fdx* promoter and NTR coding sequence were ligated into the pGG222L vector using the GGA reaction, transformed and plated on LB with erythromycin and X-gal. In the control assembly reaction, the promoter and coding sequence were replaced with an equal volume of DEPC water. The assembly that included promoter and gene amplicons (A) produced colonies that were more numerous and predominantly white compared to the control (B), which produced considerably fewer colonies that are predominantly blue. A PCR screen of eight randomly selected white colonies from the assembly plate (C) indicated that all colonies contained the correctly assembled plasmid (lanes 2–9). “L” denotes DNA ladder (NEB 1kb Plus); “V” empty vector control; “H_2_O” water control.

An overview of the workflow for assembly using the cloning and CRISPR-Cas9 vectors is given in Figure 3. To evaluate the efficiency of assembly reactions using the new vectors, a previously validated expression cassette was selected. Clostridium-directed enzyme prodrug therapy requires the heterologous expression of a nitroreductase (NTR) enzyme by tumor-colonizing *C. sporogenes*. This experimental treatment of cancer has been demonstrated previously in a nude mouse xenograft tumor model.^26^ To recreate this strain, the native sequence for the *C. sporogenes* *fdx* promoter (P*fdx*) and the codon-optimized *Neisseria meningitidis* NTR gene (reported previously) were cloned by PCR. Cloning using the type IIS restriction sites in the new vectors was sufficiently efficient to enable easy detection of correct clones. Plasmids assembled with the promoter and coding sequence fragments produced plates with an abundance of colonies that were predominantly white (Figure 4A). In contrast, control plates contained significantly fewer colonies, which were predominantly blue (Figure 4B). The relatively high number of blue colonies on control plates suggests that, in the absence of alternative clonable DNA, the MCS re-ligates to the vector backbone. Blue and white colonies from each plate were counted, and the efficiency of assembly was calculated (Table 1). Colony PCR and gel electrophoresis of white colonies (8) indicated that the screened colonies contained the correctly assembled plasmid (Figure 4C). Sanger sequencing of plasmid from a positive clone revealed correct assembly without mutation. One clone, denoted pGG222-P*fdx*-NTR, was taken forward.

**Table 1:** Colony counts for transformed GGA reactions and calculated efficiencies of assembly reactions

Assembly Reaction	Total Colonies	Blue		White		Calculated Colony Totals	
		Colonies	%	Colonies	%	Per 2ul Assembly reaction	Per Full Assembly Reaction
pGG222-Pfdx-NTR	1309	6	0.5	1303	99.5	7,993	99,914
pGG222L (Control)	75	68	90.7	7	9.3	458	5,725
pCas9n312-g7SLS-Pfdx-NTR	1265	35	2.8	1230	97.2	7,724	96,555
pCas9n312-g7SLS-GGL (control)	125	113	90.4	12	9.6	763	9,541

Colonies were counted using the ImageJ Analyze Particles feature (see STAR Methods). Colonies were counted from plates on which 160ul of recovered transformation mixture were spread following heat shock with 2ul of the assembly mixture. Calculated efficiencies are extrapolated from plate counts.

**Figure 5:**
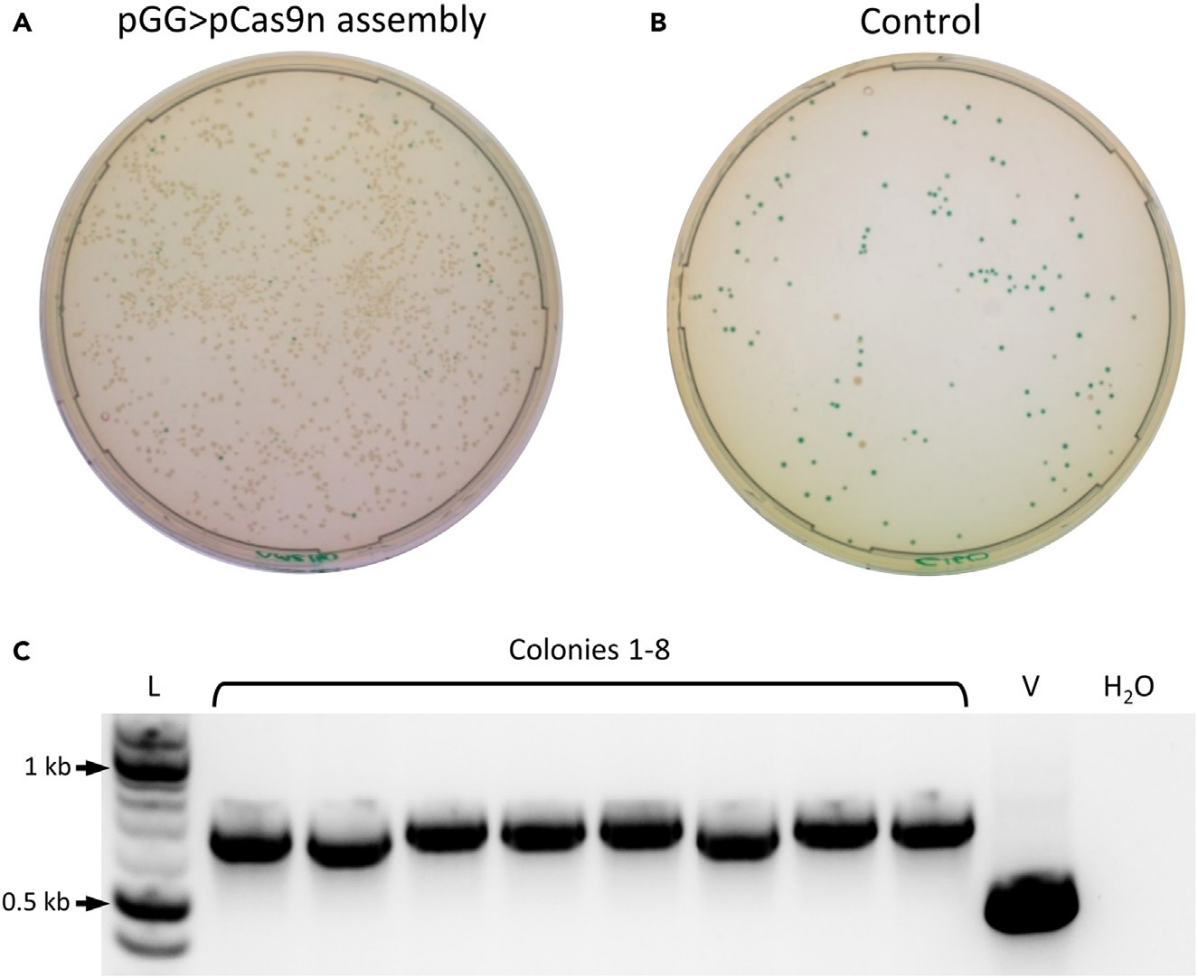
Transformation and PCR screening of the pCas9n312-g1SLS-P*fdx*-NTR assembly The subcloned P*fdx*-NTR expression cassette was transferred from the pGG222L vector to the pCas9n312-g7SLS-GGL vector using the GGA reaction, transformed and plated on LB with chloramphenicol and X-gal (A). A control assembly reaction, in which the pGG-P*fdx*-NTR purified plasmid was replaced with an equal volume of DEPC water, was conducted in parallel (B). As with the first assembly, construction of the Cas9 plasmid was sufficiently efficient to allow straightforward screening of positive colonies (white). Eight white colonies picked from the assembly plate (A) and screened by PCR produced the correct product size (C). In the control assembly reaction, the pGG-P*fdx*-NTR purified plasmid was replaced with an equal volume of DEPC water. “L” denotes DNA ladder (NEB 1kb Plus); “V” empty vector control; “H_2_O” water control.

The aim of the second assembly reaction is to exchange the *lacZα* gene of the pCas9n312-g7SLS-GGL vector for the cloned expression cassette of pGG222-P*fdx*-NTR. As the BsaI restriction sites of the pGG222L vector are excised during the first assembly reaction, the second reaction relies on the BsmBI sites that flank the MCS. Plating the resultant transformation on chloramphenicol/X-gal plates enables exclusion of two forms of background: undigested pGG222-P*fdx*-NTR (chloramphenicol-sensitive) and undigested pCas9n312-g7SLS-GGL (chloramphenicol-resistant blue colonies). This reaction is virtually identical to the first assembly, with the exception that the fragment to be cloned is precloned on the pGG222L plasmid rather than linear PCR products. Transformation and plating revealed a similar result to the first assembly: predominantly white colonies on the cloning plate and a reduced number of colonies on the control plate that were primarily blue (Figure 5A and 5B). After 24 h incubation, the colonies were noticeably smaller than those of the first assembly. In the authors’ experience this is typical, possibly due to the larger size of the vector and the constitutive expression of the Cas9 gene. A PCR screen of randomly selected white colonies indicated that assembly was correct (Figure 5C). The sequence of one colony was confirmed by Sanger sequencing and taken forward for genome integration.

### Genome integration

**Figure 6:**
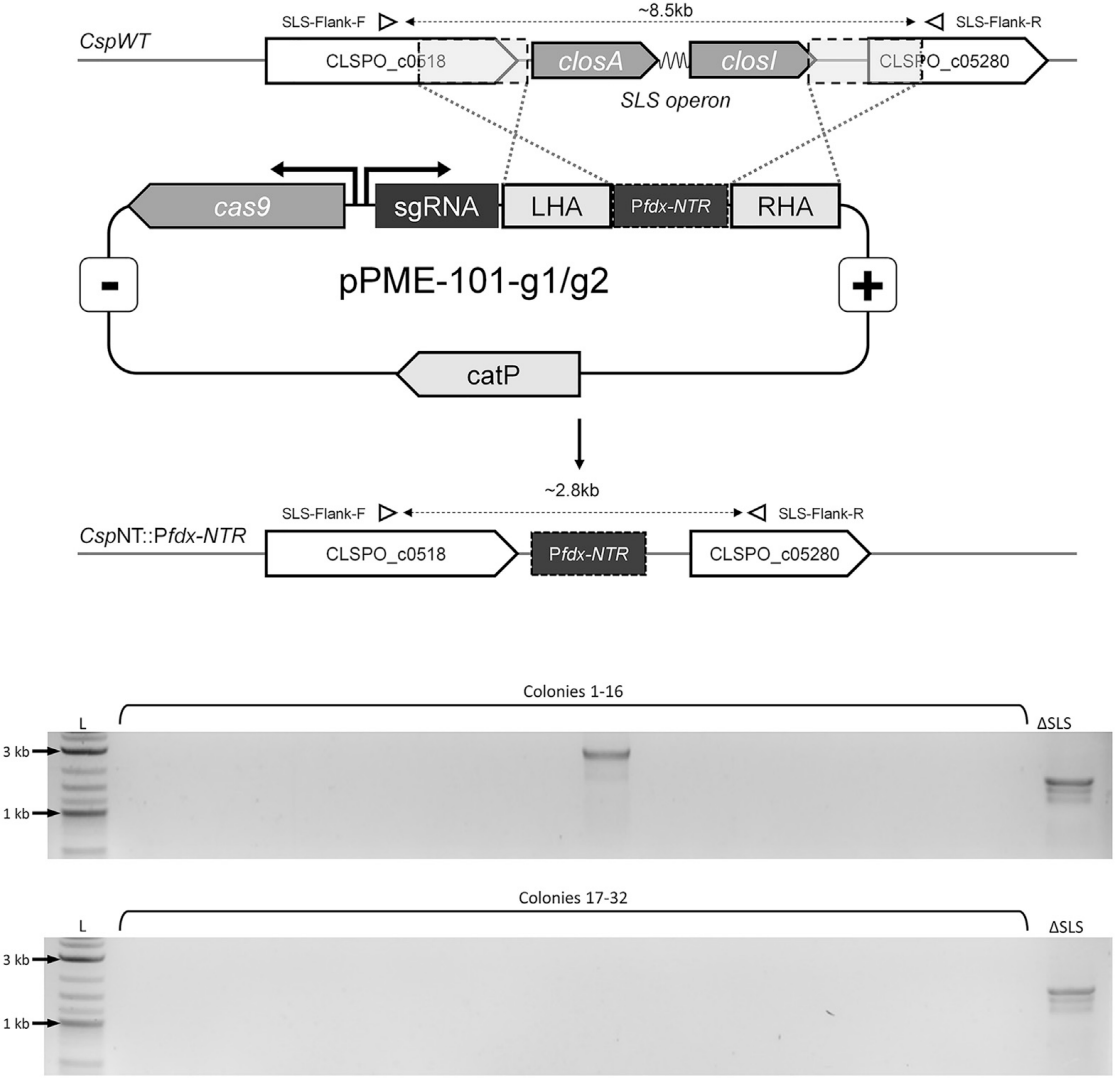
PCR screening the pCas9n312-g7SLS-P*fdx*-NTR transconjugants for P*fdx*-NTR integration at the SLS locus Primers were designed to anneal to sequences flanking the sites of homologous recombination (SLS-Flank-F and SLS-Flank-R: see Table S1). WT colonies containing the 8.5kb SLS operon do not produce a product. *C. sporogenes*-NT (“ΔSLS”), in which the SLS operon has been deleted, produces a 1.8kb product and is provided here as a control for the PCR reaction. The predicted size for P*fdx*-NTR integration is 2.8kb. Integration was detected for one colony (number 9), which was subsequently confirmed by sanger sequencing.

Genome integration of the NTR expression cassette was initially attempted with the original gRNA, used previously to delete the streptolysin S (SLS) operon homologue from *C. sporogenes*.^25^ Recombination was not detected, neither from the first conjugation selection plate nor following multiple subcultures in liquid with selection (data not shown). To improve the cutting performance of the Cas9 enzyme, the SLS operon was analyzed, using the Benchling CRISPR Guide RNA Design Tool,^27^ for target sequences that would cut more efficiently. The resultant sequence, labeled sg7, was cloned, and the integration process repeated. Following two overnight subcultures, a single integrant was detected in a PCR screen of 36 colonies (Figure 6, STAR Methods).

## Discussion

Vectors are essential for the study and exploitation of prokaryotic and eukaryotic organisms. While cloning vectors for *E. coli* have been in use since the 1970s, stable vectors for other species have taken considerably longer to develop. The publication and distribution of the pMTL8000 modular shuttle vector in 2009 were a major catalyst for the study of clostridia, a genus of bacteria that has become increasingly relevant. This publication introduces an update to these vectors that enables the use of the Golden Gate cloning method to assemble fragments directly into the plasmid. In addition, a second CRISPR-Cas9n gene editing vector has been developed to be compatible with the first vector, accelerating the creation of “knock-in” mutants. The primary objective of these changes was to make cloning and genome engineering in clostridia more accessible, with both the experienced postdoc and early PhD student in mind.

By relying solely on GGA for plasmid construction, this system can be utilized with only three enzymes: BsaI, BsmBI, and T4 DNA ligase. The gel and PCR cleanup steps required in conventional cloning are not needed, further reducing costs. While this removes the need to stock a collection of enzymes and kits, it does shift the burden to primer design. Web-based tools, such as the NEBridge Golden Gate Assembly Tool (https://goldengate.neb.com/), can assist in primer design. If the end user choses to design primers manually, particular attention must be given to the orientation of the recognition sequence to ensure that it is excluded from the fragment post-cut. It should be noted that in order for cloned fragments to be passed from the cloning vector to the gene editing vector during the assembly reaction, the BsmBI sites have opposite orientations in each vector: in the cloning vector, the recognition sites remain on the backbone, while on the gene editing vector they remain on the MCS fragment. This ensures that undesirable products (cloning vector and empty gene editing vector) are digested, while the intended assembly is not. DNA fragments can be cloned using the BsaI sites, either directly into the gene editing vector (white, Cm-resistant colonies) or via the cloning vector (white, Em-resistant). If the latter is used, the BsmBI sites must be used to transfer from cloning to gene editing vector. Both methods result in the same final expression cassette.

A number of changes were made to the parent vectors based on rational design, with the intention of improving their performance and utility. A synthetic *lacZα* replaces the MCS in both vectors to enable blue-white screening. The synthetic sequence was designed to remove any restriction sites that are present in the vector backbones to avoid issues in downstream cloning steps. The decision not to alter the promoter sequence of the *lacZα* does result in two M13R sequences in the MCS. However, this is only relevant for “empty vector” clones, which will appear blue on X-gal plates.

For the CRISPR-Cas9 vector, the Shine-Dalgarno sequence of the P*araE* promoter, present in previous versions of this vector, was deleted to avoid unwanted translation of the guide RNA, and a characterized terminator was introduced downstream of the Cas9 gene to prevent transcription readthrough into the backbone. The Rho-independent terminator of the *Lactococcus lactis pepN* gene was selected due to its documented efficiency in *C. acetobuylicum* and its small size,^24^ which enabled simple cloning as primer tails. The length of the sequence between the translation stop codon and the terminator secondary structure has been shown to affect termination efficiency.^28^ To promote efficient termination, the new terminator was incorporated 30 bp downstream of the Cas9 stop codon. The M13R primer annealing site, present downstream of the Cas9 gene in the previous vector, was not cloned to avoid an additional primer annealing site in the same vector. By cloning Cas9 in two fragments, it was possible to remove the single BsmBI restriction site in the native coding sequence. This was achieved by changing the codon encoding arginine from CGT to AGA, a preferred codon in cluster I clostridia according to recent codon usage data.^29^

In both vectors particular attention was given to creating a transcriptionally “insulated” MCS. Due to their strong secondary structure, rho-independent terminators can be challenging to clone. The hairpin can be split between adjacent fragments as primer tails during subcloning,^30^ but this is difficult to do at the junction with the vector. The terminator downstream of the MCS, inherited from the pMTL82121 vector, has been validated previously,^31^ but the upstream terminator, derived from open reading frame (ORF) CD0164 of *C. difficile* 630, has not. This was exchanged for the validated terminator from the *tyrS* gene of *Bacillus subtilis*.^24^

Vector assembly was demonstrated using the *C. sporogenes* *fdx gene* promoter and *Neisseria meningitidis* NTR gene.^26^ While GGA appeared to be highly efficient in both vectors, genome integration proved to be very inefficient. Initial attempts using a gRNA sequence that was previously used to delete the SLS locus of *C. sporogenes* failed to produce integrants. A new screen of the SLS locus yielded a new gRNA target sequence, which was cloned into the gene editing vector. Using the new construct, a single integrant was obtained from a screen of 32 colonies. This result highlights the sharp decrease in the efficiency of genome editing when “cargo” is added to the repair cassette of this system.

The motivation for developing these vectors was to support more efficient construction of plasmid- or genome-based recombinant strains and to enable standardized cloning of expression cassettes within research groups. The simplicity of GGA compared to conventional cloning opens up the possibility to clone, transform, and conjugate in microplates, as demonstrated in an equivalent system for *Bacteroides* species.^32^ This must be designed carefully in order to downscale volumes while preserving reactions kinetics. However, the creation of these vectors and the demonstration of a 96-well workflow in *Bacteroides* (which also relies on conjugative transfer from *E. coli*) make it a considerably easier task. The introduction of these vectors, which are openly available to all researchers, will enable high-throughput experiments that can yield large datasets, and it is our hope that this will accelerate the study of *Clostridium* biology. In addition, the design of these vectors provides a concept that can be adopted and adapted to any other species, promoting a smarter, more refined method of strain development.

### Limitations of the study

The vectors in their current format can be used in bacteria that support the gram-positive or gram-negative replicons present on the vector backbone. The segregational stability of the gram-positive replicons, along with several others, has been reported previously. Depending on the species of *Clostridium* in which the vectors will be used, the replicon will need to be changed according to the reported data.^33^^,^^34^ For the CRISPR-Cas9 vector, users should avoid using very stable replicons, which may lead to issues with plasmid loss following genome editing. The CRISPR-Cas9 vector is currently designed to target the SLS operon of *C. sporogenes* NCIMB 10696. The current homology arms and gRNA must be changed in order to target genome loci in other *Clostridium* species. The MCS can be retained using the MCS-intBsmBI-F/MCS-intBsmBI-R primer pair (See Table S1) and cloned conventionally using the BsmBI sites.

The major limitation of the current vectors is the apparent reduced potency of the Cas9 nickase, in contrast to the original, unaltered spCas9. In the current vector design, both the Cas9n gene and the gRNA are expressed constitutively. The promoter driving the Cas9 gene, P*thl*, was recently shown to produce very high levels of gusA reporter activity in *E. coli*, but low levels in *C. sporogenes* and *C. butyricum*.^35^ This suggests that the truncation of the Cas9 gene to a nickase, as reported previously,^13^ is likely to have occurred during cloning in *E. coli*, possibly due to the inherent toxicity of the Cas9 protein.^36^ Utilizing the true spCas9 in the gene editing vector is likely to require an inducible promoter for the Cas9 gene, and possibly the gRNA too.

## STAR★Methods

### Key resources table

**Table:** 

REAGENT or RESOURCE	SOURCE	IDENTIFIER
**Bacterial and virus strains**		

*E. coli* 10-beta	New England Biolabs Inc.	C3019H
*E. coli* s17-1	DSMZ GmbH	9079
*C. sporogenes*	NCIMB (UK)	NCIMB 10696

**Oligonucleotides**		

Oligonucleotides	Integrated DNA Technologies	Custom DNA oligos, LabReady, Standard Desalting (No modifications)

**Recombinant DNA**		

lacZα	Genewiz (Azenta Life Sciences)	Gene Synthesis ("Gene Fragments" service)

### Resource availability

#### Lead contact

Further information and requests for resources and reagents should be directed to and will be fulfilled by the lead contact, Tom Bailey (t.bailey@maastrichtuniversity.nl).

#### Materials availability

Plasmids generated in this study have been deposited to Addgene and data files have been submitted to NCBI GenBank under the following details:

pGG222L - Addgene ID 196994, GenBank accession number OQ302270.
pCas9n312-g7SLS-GGL: Addgene ID 197042, GenBank accession number OQ302271.

### Experimental model and subject details

#### Bacterial strains, media and culture conditions

Liquid cultures of *E. coli* strains were grown in LB broth (10 g/l tryptone, 5 g/l yeast extract, 10 g/l sodium chloride) at 37°C with shaking (200rpm). Overnight cultures were grown in 30ml universal tubes. Subcultures were grown in erlenmeyer flasks, filled to a maximum of 1/3 total capacity. Solid media for plate growth was produced by addition of 2 % (w/v) agar to LB broth prior to autoclaving. Plate cultures were incubated at 37°C (static). When required, chloramphenicol (Cm) (15 μg/ml), erythromycin (Em) (250 μg/ml), D-cycloserine (cyc) (250μg/ml) and X-gal (200 μg/ml) were added to molten agar prior to pouring plates. *C. sporogenes* was grown in TY medium supplemented with sodium thioglycolate (30 g/l tryptone, 20 g/l yeast extract, 1 g/l sodium thioglycolate), adjusted to pH 7.4 prior to autoclaving. Solid media was produced by addition of 2 % (w/v) agar prior to autoclaving. Cultures were incubated at 37°C under anaerobic conditions (80% N_2_, 10% CO_2_, 10% H_2_) in a Don Whitley Scientific anaerobic workstation (model DG250). Details of all bacterial strains used in this study are listed in Table 1.

### Method details

#### Vector construction

Details of the primers and synthetic DNA used in vector construction are provided in Table S1. A schematic of the construction process for the cloning and gene editing vector is presented in Figures 1 and 2, respectively. When designing primers that included restriction sites, additional bases were included after the recognition site, according to the manufacturer’s guidelines.^37^ Final vectors were confirmed by complete sanger sequencing.

The first step in construction followed the method described previously, but used vector pMTL82221 as template. This vector was digested MreI / NheI and the resultant 4.4 kb backbone gel purified. Using primer pairs BsmBI-MreI-F / BsmBI-TtyrS-R and BsmBI-MCS-F / BsmBI-MCS-R and pMTL82221 as template, two PCR fragments were created. Both fragments were BsmBI digested and ligated to the digested backbone, resulting in vector “pMTL8222G”. This was digested MreI/FseI, and the resultant 1.7 KB was gel purified. Using pMTL8222G as template, three PCR fragments were created using primer pairs BsmBI-MreI-F / traJ-R, traJ-F / pBP1-R and pBP1-F / FseI-R, respectively. These fragments corresponded to a fragment upstream of the multiple cloning site (MCS), the MCS itself, and a fragment downstream of the MCS to the FseI restriction site. The PCR fragments were digested (BsmBI) and ligated with the MreI / FseI fragment (NEB T4 DNA ligase, manufacturer’s protocol). The resultant ligation mixture was heat shock transformed into *E. coli* 10-beta competent cells (NEB product C3019H, manufacturer’s protocol) and screened using primer pair pGG-F/R. Correct clones (“pGG222”) were confirmed by sanger sequencing.

A synthetic *lacZα* expression cassette was designed and synthesised commercially (Genewiz “fragmentGENE”, see Table S1 for sequence). The native *E. coli lacZα* sequence was altered using alternative codons in order to eliminate commonly used restriction sites, including those flanking the backbone modules. BsaI restriction sites, designed to remain on the MCS fragment after cutting, were added at the 5’ and 3’ end of the sequence. pGG222 was BsaI digested and the backbone purified by gel electrophoresis. The synthetic *lacZα* was BsaI digested and annealed to the backbone by conventional ligation.

The Cas9 gene editing vector was derived from the plasmid reported previously.^25^ Five fragments were created by PCR using this plasmid as template: the Cas9 gene and upstream divergent promoters in two fragments (primer pairs pepN-Cas9-F / Cas9-delBsmBI-R and Cas9-delBsmBI-F / ParaE-ΔRBS-R), the guide RNA (gRNA) and left homology arm (LHA) (SLSg1-SalI-F / sagLHA-BsmBI-R), the right homology arm (RHA) (sagRHA-BsmBI-F / sagRHA-AscI-R) and the remaining backbone (sagRHA-AscI-F / pepN-traJ-R (BsmBI)). The MCS, designed to be ligated between the homology arms, was derived from pGG222L using primers MCS-intBsmBI-F / MCS-intBsmBI-R. The resultant six fragments were digested and ligated overnight using a ratio of 3 for the small fragments (5’ Cas9-ParaE, gRNA-LHA, MCS, RHA) to 1 for the backbone and 3’ Cas9 fragments. The ligation was transformed as for the cloning vector (see above).

A second version of the vector was created with a new gRNA target sequence. The new gRNA was created using primers SLSg7-SalI-F / gRNA-R with the vector as template and digested SalI / AatII. The original gRNA was excised from the vector by SalI / AatII digestion, and the resultant backbone was ligated to the new gRNA fragment at 1:5 ratio.

#### Plasmid assembly and transformation

An overview of the assembly process is given in Figure 3. To demonstrate GGA, the promoter and coding sequence of the previously characterised P*fdx*-NTR expression cassette were PCR cloned as two fragments using primer pairs Pfdx-F / Pfdx-R and NTR-F / NTR-R. GGAs using vector pGG222L were carried out according to the NEB protocol for amplicon cloning with the following modifications. Due to the high efficiency of commercial competent cells, half of the provided volume (25 μl) was used for each transformation, to which 2ul of the GGA reaction was added. Following heat shock transformation and recovery, three different volumes of cell suspension were plated on selection plates: 80 μl, 160 μl and the remaining volume (condensed by centrifugation and resuspension in 200 μl). The second assembly reaction was carried out according to the NEB protocol for precloned inserts, with particular attention paid to the 2:1 molar ratio. Transfomation was conducted as for the first assembly. For control assemblies, an equal volume of water was substituted for the amplicon or precloned fragment. Colonies formed after transformation were counted using the Fiji distribution of ImageJ.^38^ Briefly, images of agar plates were masked so that only the agar surface was visible. Images were converted to 8-bit, segmented using thresholding and colonies counted using the “Analyze Particles” feature. The process was repeated with a lower threshold to distinguish the darker blue colonies from the white colonies, in order to quantify blue colonies alone. Total colony counts for the whole transformation mixture (950 μl recovery media, 25 μl competent cells, 2 μl assembly reaction) were calculated using the formula: [Calculated colony total (2 μl assembly reaction) = (colony count/160)∗977]. The potential total colony count for the whole assembly reaction (25 μl) was extrapolated by multiplying the calculated total for 2μl by 12.5.

#### CRISPR-Cas9 genome editing

Following sanger sequencing, the purified pCas9n312-g7SLS-P*fdx*-NTR plasmid was heat shock transformed into conjugation strain *E. coli* s17-1 and plated on Cm selection plates. After overnight incubation, a single colony was picked to Cm selection broth. On the same day, a *C. sporogenes* liquid culture (no selection) was setup from plate growth, streaked the day before. Following overnight incubation, 1 ml of s17-1 cultures were centrifuged (5000 *x g*, 5 minutes), gently washed with PBS, centrifuged again and the supernatant removed. The resultant pellet was resuspended with 200 μl of the *C. sporogenes* culture then spotted on to TY plates without selection. After 7 h anaerobic incubation, the spots were removed from the surface of the agar using a large inoculation loop and resuspened in 500 μl PBS. Two different volumes (80 and 160 μl) of this suspension was then spread on two separate TY/Cyc/Cm plates. After 24-48h incubation, Cm-resistant *C. sporogenes* colonies formed on the selection plates. These were picked and pooled in 10 ml TY/Cyc/Cm broth and incubated overnight. The resultant culture was subcultured again before serial dilution and plating of the 10-5 and 10-6 dilutions (200 μl). Colonies that formed after overnight incubation were screened by PCR using primers SLS-Flank-F and SLS-Flank-R, which flank the SLS locus.

## Data Availability

• All data reported in this paper will be shared by the lead contact upon request. • This paper does not report original code. • Any additional information required to reanalyze the data reported in this paper is available from the lead contact upon request.
