# Supplementary material for: Streamlined assembly of cloning and genome editing vectors for genus Clostridium
Source: iScience. 2023 Jul 28;26(8):107484. doi: 10.1016/j.isci.2023.107484 (PMC10432817; doi:10.1016/j.isci.2023.107484)
Supplement: Document S1. Table S1 [file mmc1.pdf]

iScience, Volume *26*

Supplemental information

Streamlined assembly of cloning and genome editing vectors for genus *Clostridium*

Tom S. Bailey, Philip Hittmeyer, Yanchao Zhang, and Aleksandra M. Kubiak

Table S1: Primers and recombinant DNA used for construction of vectors pGG222L and pCas9n312-g7SLS-GGL, related to figures 4, 5 and 6, and the STAR methods. Primers were used for vector construction, PCR screening and sequencing vectors and genome modifications. The third column of the table details in which process the primer was used. See the STAR methods section for more detail.

Primer	Sequence	Description
BsmBI-MreI-F	TATACGTCTCACCGGCGTAACAGATGAGGGC	Cloning pMTL8222G (pMTL82221 template) and pGG222 (pMTL8222G template)
BsmBI-TtyrS-R	TATACGTCTCAACACGAAGGGACGATTGATTGATTATCCTGCAGGGGGCCC	Cloning pMTL8222G (pMTL82221 template)
BsmBI-MCS-F	TATACGTCTCTGTGTAAACGAAGGGGCGTTTTTTATTTCAGGAAACAGCTATGACCGCGGCCGCTGTATCCATGAGACC	Cloning pMTL8222G (pMTL82221 template)
BsmBI-MCS-R	TATACGTCTCGCTAGTGTAAAACGACGGCCAGTGCCAAGCTTGCATGTCTGGAGACCTCG	Cloning pMTL8222G (pMTL82221 template)
traJ-R	TCCAGGAGACGCGATCGGTCTTGCCTTGC	Cloning pGG222 (pMTL8222G template)
traJ-F	CGTCTCCTGGAGGATAATCAATCGTCCC	Cloning pGG222 (pMTL8222G template)
pBP1-R	CGTCTCTTCAGAACGGCGCGCCAT	Cloning pGG222 (pMTL8222G template)
pBP1-F	CCGTTCTGAAGAGACGGCTAATGG	Cloning pGG222 (pMTL8222G template)
FseI-R	TTTTCAACTTGCCCACTGGCCG	Cloning pGG222 (pMTL8222G template)
pepN-Cas9-F (BsmBI)	CGTCTCTAGAGGTGGTTTTTTTATTTATAAATTACTGTATCCATATGACCATGATTACGAATTC	Cloning pCas9-SLS-GG (pPME-101-g1 template)
Cas9-ΔBsmBI-R	TGGAGAGACAGCGGAAGCG	Cloning pCas9-SLS-GG (pPME-101-g1 template)
Cas9-ΔBsmBI-F (BsmBI)	CGTCTCCCGTTTGAGTCTAGTCGC	Cloning pCas9-SLS-GG (pPME-101-g1 template)
ParaE-ΔRBS-R	TACGGTCGACTAAGATTTATATATGTGGAAATCAAAGAGAAATC	Cloning pCas9-SLS-GG (pPME-101-g1 template)
SLSg1-SalI-F	AGAGTCGACGAGTTAATCCATCTGCAGG	Cloning pCas9-SLS-GG (pPME-101-g1 template)
SLSg7-SalI-F	TTAGTCGACGCAGTACCTGTACTAAAGGAGTTTTAGAGCTAGAAATAGCAAG	Cloning pCas9-SLS-GG (pPME-101-g1 template)
gRNA-R	CCGACGTCATAAAAATAAGAAGCCTGCAAATGCAG	Cloning pCas9-SLS-GG (pPME-101-g1 template)
sagLHA-BsmBI-R	CGTCTCTTCCACCTCAAATTTATTATACCTAATAATCC	Cloning pCas9-SLS-GG (pPME-101-g1 template)
MCS-intBsmBI-F	TTTCCATGAGACCAAAGCGGGCAGTGAGCG	Cloning pCas9-SLS-GG (pPME-101-g1 template)
MCS-intBsmBI-R	TTGTCTTGAGACCTTATGCGGCATCAGAGCAGATTG	Cloning pCas9-SLS-GG (pPME-101-g1 template)
sagRHA-BsmBI-F	CGTCTCGCTGACCTTAGTATAGTAATTTTATTTGCAGTAAG	Cloning pCas9-SLS-GG (pPME-101-g1 template)
sagRHA-AscI-R	GGCGCGCCCCAAAGTTCACTAATTTTAAC	Cloning pCas9-SLS-GG (pPME-101-g1 template)
sagRHA-AscI-F	AGTGAACTTTGGGGCGCG	Cloning pCas9-SLS-GG (pPME-101-g1 template)
pepN-traJ-R (BsmBI)	CGTCTCACTCTAAAAGGTGATTTTTATTTATAAATTATCTTGCCTTGCTCGTCGGTG	Cloning pCas9-SLS-GG (pPME-101-g1 template)

pGGv2-F	CCGATCGCGTCTCCTGG	PCR screening and sequencing pGG222L
pGGv2-R	CTGTTGAACCATTAGCCGTCTC	PCR screening and sequencing pGG222L
SLSg7-SalI-F	TTAGTCGACGCAGTACCTGTACTAAAGGAGTTTTAGAGCTAGAAATAGCAAG	Cloning SLS gRNA 7
SLS-Flank-F	TAGCATCAGGAGGAACGAAGATAAAGGC	Screening for genome integration at the SLS locus
SLS-Flank-R	CCATAAATCTCTCAATATGTCAAAGCCATCAAGTCC	Screening for genome integration at the SLS locus
Pfdx-F (BsaI)	GGTCTCGTCCATATAAAAATTACTTTAAAAATTAATAAAAACATGGT	Cloning *fdx* gene promoter from *C. pasteurianum*
Pfdx-R (BsaI)	GGTCTCGTCATATGTAACACACCTCCTTAAAAAT	Cloning *fdx* gene promoter from *C. pasteurianum*
NTR-F (BsaI)	GGTCTCGATGACAGTATTATCTAAAGAACAAGTATTATC	Cloning the codon optimised nitroreductase gene of *Neisseria meningitidis* MC58 (see Heap *et al.* 2014)
NTR-R (BsaI)	GGTCTCAGTCTTTAAGCCCATATTACAGTCTCTTC	

**Recombinant DNA**	**Sequence**	**Description**
lacZα	TCCATGAGACCAAAGCGGGCAGTGAGCGCAACGCAATTAATGTGAGTTAGCTC ACTCATTAGGCACCCCAGGCTTTACACTTTATGCTTCCGGCTCGTATGTTGTGTG GAATTGTGAGCGGATAACAATTTCACACAGGAAACAGCTATGACCATGATTAC GCCAAGCTTGCATGCCTGCCGGTCCACGCTAGAGGATCCCCGGGTACCGAGCT CGAATTCATTAGCTGTTGTGTTGCAACGTCGTGACTGGGAAAACCCTGGCGTTA CCCAACTTAATCGCCTTGCAGCACATCCCCCTTTCGCCAGCTGGCGTAATAGCGA AGAGGCCCGCACCGATCGCCCTTCCCAACAGTTGCGCAGCCTGAATGGCGAAT GGCGCCTGATGCGGTATTTTCTCCTTACGCATCTGTGCGGTATTTCACACCGCAT TTGGTGCACTCTCAGTACAATCTGCTCTGATGCCGCATAAGGTCTCAAGAC	Synthetic sequence of the lacZα promoter and coding sequence. Using alternative codons, the coding sequence was altered to remove restriction sites. The sequence is flanked by BsaI sites oriented towards the termini to allow conventional restriction cloning into the pGG222 vector.
